# Whole-brain 3D MR fingerprinting brain imaging: clinical validation and feasibility to patients with meningioma

**DOI:** 10.1007/s10334-021-00924-1

**Published:** 2021-05-04

**Authors:** Thomaz R. Mostardeiro, Ananya Panda, Robert J. Witte, Norbert G. Campeau, Kiaran P. McGee, Yi Sui, Aiming Lu

**Affiliations:** Department of Radiology, Mayo Clinic, Rochester, MN USA

**Keywords:** Whole-brain MR fingerprinting, Meningioma, Relaxometry, 3D isotropic

## Abstract

**Purpose:**

MR fingerprinting (MRF) is a MR technique that allows assessment of tissue relaxation times. The purpose of this study is to evaluate the clinical application of this technique in patients with meningioma.

**Materials and methods:**

A whole-brain 3D isotropic 1mm^3^ acquisition under a 3.0T field strength was used to obtain MRF T_1_ and T_2_-based relaxometry values in 4:38 s. The accuracy of values was quantified by scanning a quantitative MR relaxometry phantom. In vivo evaluation was performed by applying the sequence to 20 subjects with 25 meningiomas. Regions of interest included the meningioma, caudate head, centrum semiovale, contralateral white matter and thalamus. For both phantom and subjects, mean values of both T_1_ and T_2_ estimates were obtained. Statistical significance of differences in mean values between the meningioma and other brain structures was tested using a Friedman’s ANOVA test.

**Results:**

MR fingerprinting phantom data demonstrated a linear relationship between measured and reference relaxometry estimates for both T_1_ (*r*^2^ = 0.99) and T_2_ (*r*^2^ = 0.97). MRF T_1_ relaxation times were longer in meningioma (mean ± SD 1429 ± 202 ms) compared to thalamus (mean ± SD 1054 ± 58 ms; *p* = 0.004), centrum semiovale (mean ± SD 825 ± 42 ms; *p* < 0.001) and contralateral white matter (mean ± SD 799 ± 40 ms; *p* < 0.001). MRF T_2_ relaxation times were longer for meningioma (mean ± SD 69 ± 27 ms) as compared to thalamus (mean ± SD 27 ± 3 ms; *p* < 0.001), caudate head (mean ± SD 39 ± 5 ms; *p* < 0.001) and contralateral white matter (mean ± SD 35 ± 4 ms; *p* < 0.001)

**Conclusions:**

Phantom measurements indicate that the proposed 3D-MRF sequence relaxometry estimations are valid and reproducible. For in vivo, entire brain coverage was obtained in clinically feasible time and allows quantitative assessment of meningioma in clinical practice.

## Introduction

Magnetic resonance fingerprinting (MRF) is a novel MR acquisition method for quantitative assessment of tissue magnetic properties such as T_1_, T_2_ and proton density [1–3]. Introduced in 2013, MRF involves acquiring either a two or three-dimensional dataset typically using non-Cartesian k-space encoding sampling scheme such as a spiral trajectory [2, 4, 5]. Unlike conventional acquisitions which commonly require establishment of a steady state of the magnetization before spatial encoding, a MRF pulse sequence modifies acquisition parameters, including the radiofrequency flip angle, the pulse repetition rate (TR) and echo time (TE), over a time interval while continuously acquiring data [6–8]. For a given voxel in the reconstructed MR images and given that the acquisition parameters are known, a simulated or equivalent MR signal time course can be generated for a given set of relaxometry values [9–11]. For each voxel, the signal evolution obtained is compared with a collection of simulated signal evolutions (or fingerprints). The best match for the voxel fingerprint is selected from this collection (the dictionary) through a pattern matching process. Processing of all voxels in this manner results in the generation of quantitative spatial relaxometry maps [10, 11], thereby providing a method to assess the underlying magnetic properties of the tissue in both normal and disease states [11–14].

Phantom experiments have validated the accuracy and precision of MRF relaxometry estimates [8, 11, 14–16using a quantitative relaxometry phantom developed jointly by the National Institute of Science and Technology (NIST) and the International Society of Magnetic Resonance in Medicine (ISMRM) [15] as well as agarose gel [12, 17, 18] and T1MES [19] phantoms. Also, under in-vivo neuroimaging, MRF relaxometry has been demonstrated to be repeatable and reproducible across multiple scanners [20] and field strengths [21]. Clinically it has also been demonstrated that 2D-MRF-based relaxometry can identify abnormalities that are poorly visualized using conventional MR imaging in patients with epilepsy [7, 22]. In addition, slice selective 2D-MRF has been proposed for differentiating intra-parenchymal brain tumors, such as: high-grade gliomas [23, 24] (World Health Organization grades III and IV), low-grade gliomas [23, 24] (World Health Organization grades I and II) and metastases [23]. Given the mentioned investigations in neuroimaging [22–24] studied only selected 2D-MRF slices of the brain, 3D-MRF has been proposed [5, 25–27] to allow a fast whole-brain coverage in a Radiation Oncology setting [25] and in patients with Parkinson Disease [28]. These studies [25, 28] demonstrated that a fast entire brain coverage was feasible with high resolution, pointing a major advantage of a 3D-MRF acquisition for clinical applications.

Meningioma are classified by the World Health Organization into three grades: grade I (benign), grade II (atypical meningioma), and grade III (anaplastic or malignant meningioma) [29], accounting for 13–26% of intracranial tumors with the vast majority (85%) being benign [29, 30]. Quantitative imaging techniques have been proposed for tissue characterization in meningioma, such as ADC values [31–33] and MR elastrography [34]. This pilot investigation has two goals. First, it aims to further validate the relaxometry estimations from a whole-brain 3D-MRF [9] acquisition through a NIST/ISMRM phantom experiment. Second, it aims to evaluate the clinical feasibility of this sequence into a clinical cenario by providing further insight for relaxometry properties in charaterization of meningiomas and selected brain structures.

## Methods

### Image acquisition and reconstruction

All exams were acquired on two 3T clinical MR scanners (Discovery MR750 and Discovery MR750W, GE Healthcare, Waukesha, WI) using an eight channel receive-only RF head coil. MRF data acquisition was performed using a three-dimensional steady state free precession sequence with a novel multi‐axis spiral trajectory and slab excitation [9]. Adiabatic inversion pulses were used before each acquisition. A ramp flip angle schedule was used ranging from 0.778° to 70°. The sequence details can be found in [9]. A volumetric k-space data set consisting of 256 × 256 × 256 samples and a FOV of 25.6 × 25.6 × 25.6 cm^3^ resulted in reconstructed relaxometry maps with 1 mm isotropic resolution. The total acquisition time for the whole brain was 4:38 (minutes: seconds). The T_1_ for the dictionary ranged from 10 to 3000 ms and T_2_ from 10 ms up to 2000 ms. Each T_1_ and T_2_ dictionary step range follow the exponential nature of T_1_ and T_2_ relaxation curves. For example, 10:5:100 means step size 5 range from 10 to 100 ms. As such, a few examples for T_1_ curve steps are 10:5:100, 110:10:1000, 1050:50:2000, 2100:100:3000 and for T_2_ = 10:1:100, 105:5:500, 525:25:1000, 1100:100:2000. Fingerprint reconstruction and dictionary matching were performed offline in Matlab (Mathworks, Natick, Massachusetts) on a Linux workstation equipped with two 8‐core Intel Xeon Gold 6244 central processing unit @ 3.60 GHz, 376 GB system memory, and a NVIDIA Tesla V100 graphical processing unit. The reconstruction pipeline has been described elsewhere [35]. Briefly, the undersampled data were anti-aliased with a k-space-weighted view-sharing algorithm and [36, 37] then the view-shared data were compressed with singular value decomposition algorithm and finally the first 15 singular value decompensation coefficients of the temporal signals were kept for parametric maps reconstruction. GPU gridding and fast Fourier transform were performed on the compressed non-Cartesian k-space data. T_1_, T_2_ and proton density maps were computed via dictionary matching on the general processing unit and interpolated to 512 × 512 × 512 image matrix for display. The computation time was approximately 10 min for each dataset.

### Phantom validation

To evaluate the accuracy and reproducibility of the MRF-based relaxometry maps, a quantitative MR relaxometry phantom developed jointly by the NIST and ISMRM [38] was scanned 7 times over the course of 30 min. This phantom has compartments containing solutions with a wide range of T_1_ and T_2_ values [15]. MRF-based T_1_ and T_2_ values were compared with the calibrated nominal values provided with the phantom.

### Patient studies

An institutional review board approved study was used to acquire MRF data in patients scheduled for a diagnostic brain MR examination. The MRF sequence was added to the clinical protocol and acquired prior to the administration of a gadolinium-based contrast agent to assess the native relaxometry of the in-vivo brain.

**Fig. 1:**
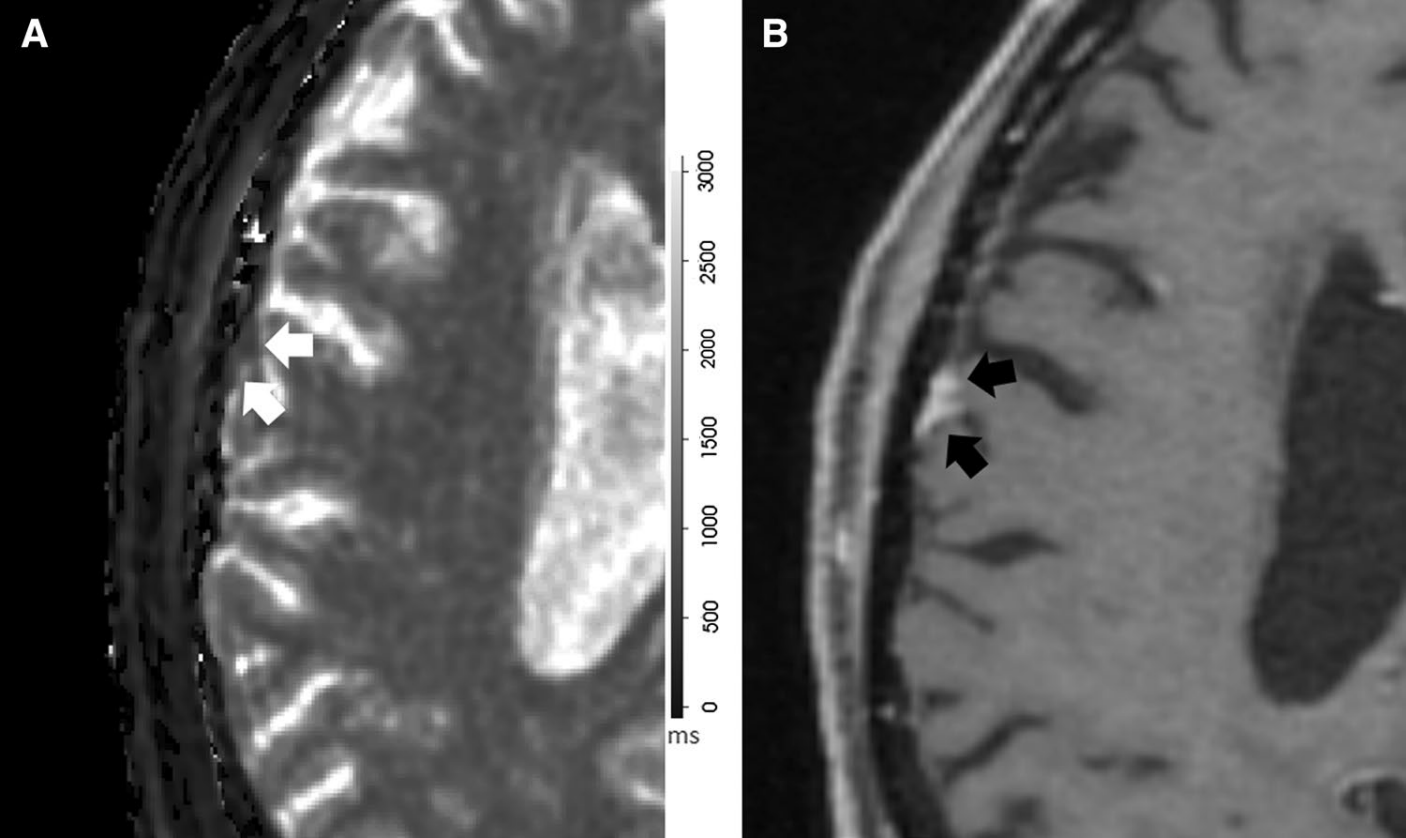
Anatomic and MRF data of subject excluded due to partial volume effects. **a** MRF T_1_-relaxometry map shows a small dural-based right parietal meningioma overlying the right operculum with the lowest T_1_ value (793 ms). This was also the smallest lesion (0.9 cm) in this study. **b** Axial spin echo post-gadolinium T_1_WI shows typical homogeneous enhancement and extradural location of meningioma

Only patients with biopsy-proven or imaging diagnosis of meningioma identified on prior diagnostic MRI examinations were included. Radiation induced meningioma (RIM) were included but those that were treated with gamma knife stereotactic radiosurgery were excluded. Subject ages ranged from 18- to 76-years old. A total of 22 patients were recruited with two being excluded from the statistical analysis. The first patient was excluded due to the small size of the mass and the effect of partial volume averaging (Fig. 1) on relaxometry values while the second was excluded due to concerns about being a potential extradural metastasis instead of meningioma on expert neuroradiology re-review. The final population comprised twenty patients; four patients had multiple tumors thus totaling 25 meningiomas. Seventeen tumors were non-treated meningiomas (NTM), four partially resected meningiomas (PRM) and four prior brain-radiation-induced meningiomas (RIM). Pathology was available for nine meningiomas (3 = Grade I; 6 = Grade II) in different groups: two RIM, three NTM and all four PRM. For NTM with biopsy results available, the MRF scan was done before biopsy to avoid tract changes. PRM were included only if the residual tumor was clearly defined without any described operative change.

### MRF image analysis

**Fig. 2:**
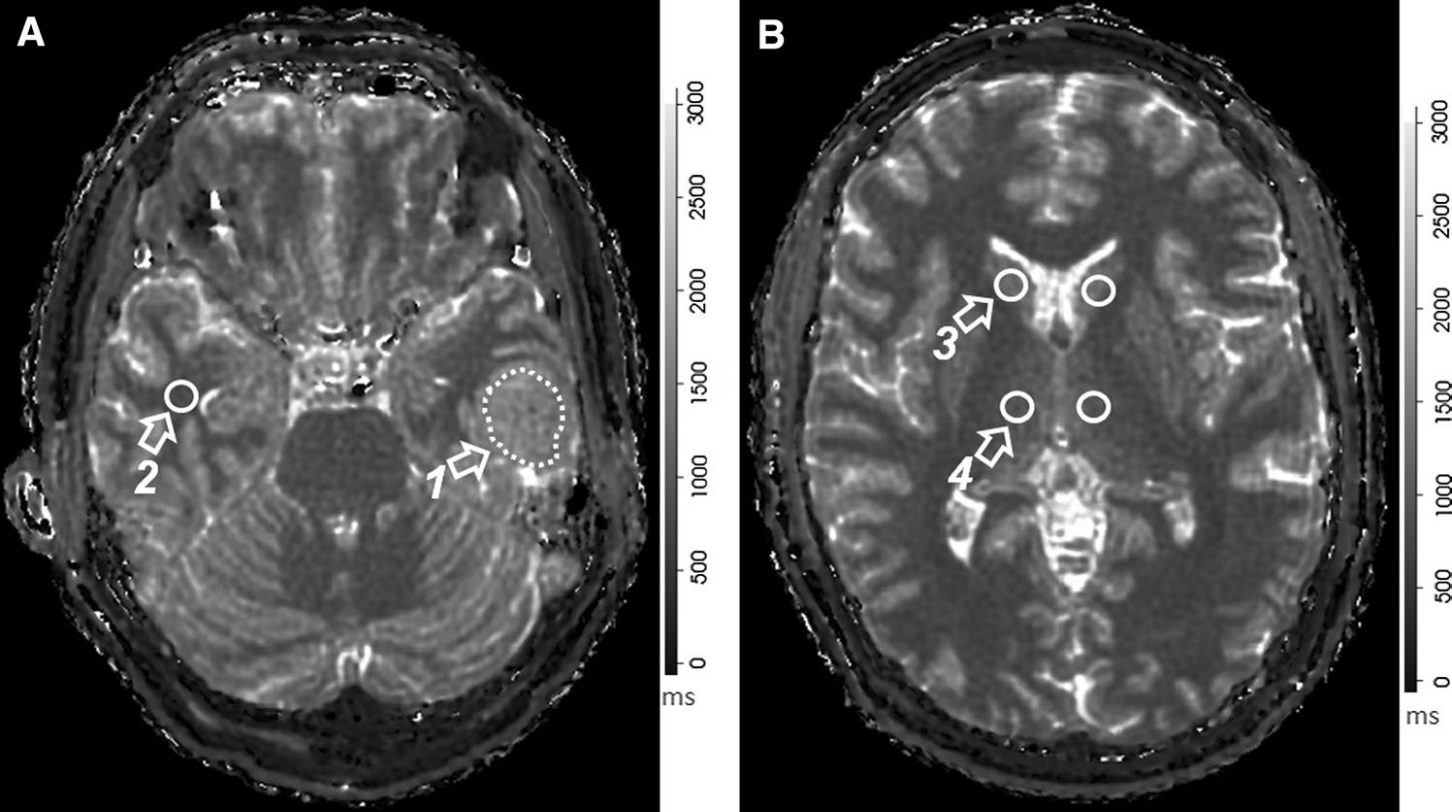
MRF-derived T_1_ relaxation maps from the same subject. **a** Depicts ROI drawings in the (1) meningioma and (2) contralateral white matter (CWM). **b** Illustrates the ROIs used to encompass the caudate head (CH) (3) and thalamus (4). The grayscale is calibrated in milliseconds (ms), shown to the right of the respective relaxation maps

To estimate in-vivo brain relaxometry values, regions of interest (ROI) were drawn directly over T1 MRF relaxometry maps using the ITK-SNAP software Version 3.8 [39]. ROIs were then copied to MRF T_2_ maps. Clinical pre- and post-contrast MRI images were used as reference for all 15 study patients. ROIs were drawn to include the meningioma as well as normal structures that included the centrum semiovale (CS), thalamus, contralateral white matter (CWM) and caudate head (CH). The rationale in selecting normal brain structures that are both on deep gray matter and white matter is to compare how the MRF numbers we described on those structures are in accordance to more established literature descriptions on relaxometry in these same structures. All ROI were reviewed by board-certified neuroradiologists prior to finalization. Figure 2 illustrates ROIs used to quantify these regions. If the meningioma crossed the midline, then the CWM ROI in the hemisphere opposite to the epicenter of the lesion was chosen. In those patients who had more than one meningioma, the location of the CWM ROI was chosen in the hemisphere opposite the largest lesion.

### Statistical analysis

To assess the precision and accuracy of MRF-based phantom relaxometry values, a linear regression analysis (Microsoft Excel 2010, Microsoft Corporation, Redmond, WA) was performed on the MRF phantom data. Repeatability was calculated by measuring the standard deviation of the phantom values over the seven repeat acquisitions obtained over the course of the 30-min imaging session.

The means of the MRF-based T_1_ and T_2_ values for meningioma and normal brain structures were compared using a nonparametric Friedman’s Test ANOVA (OriginPro 2020b, Northampton, MA) that included a post-hoc Dunn’s analysis to determine statistical significance between ROI. In patients with multiple tumors, each meningioma was paired multiple times with normal brain structures. A *p* value < 0.05 was used for statistical significance.

## Results

### Phantom

**Fig. 3:**
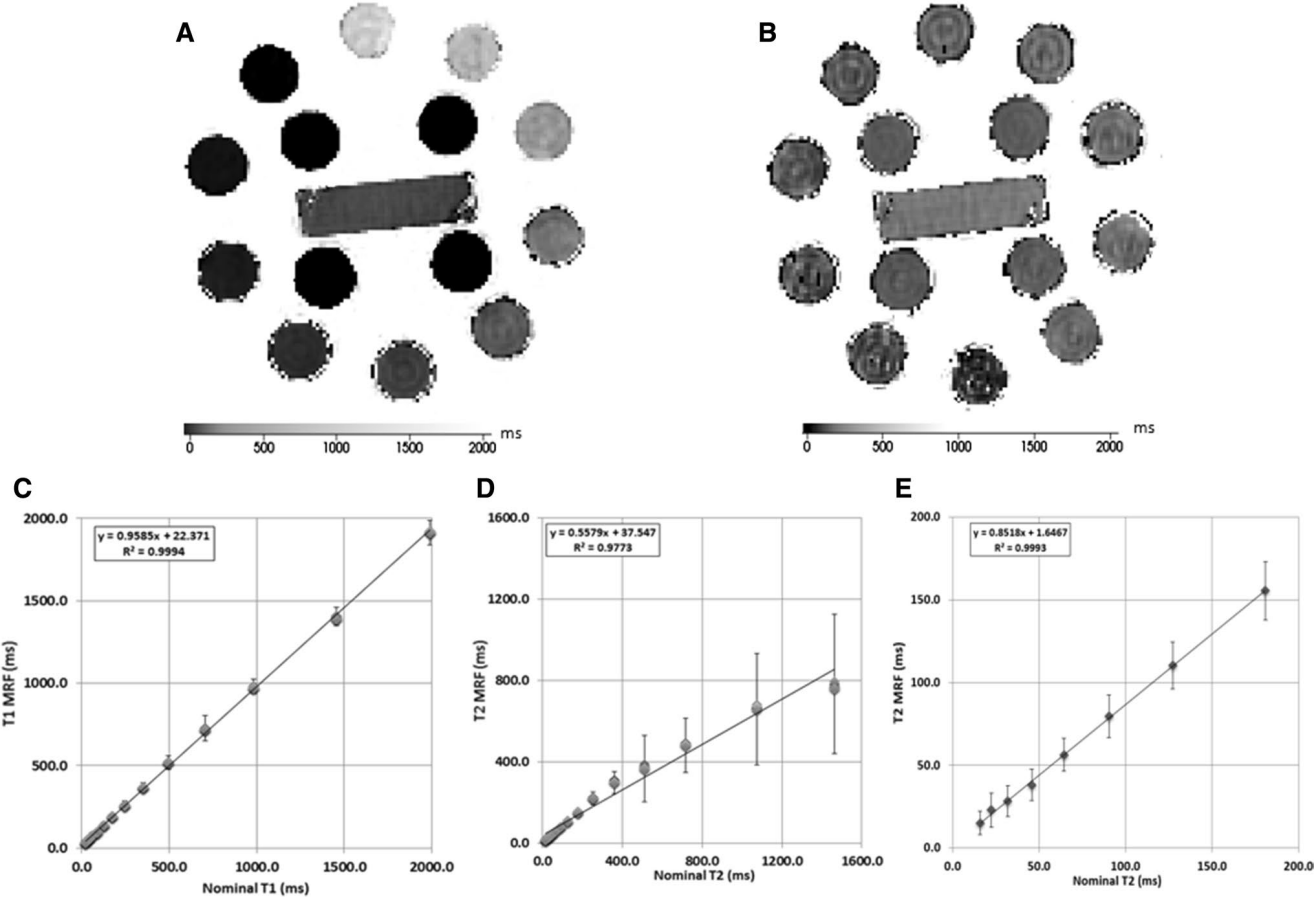
Phantom validation. **a**, **b** T_1_ relaxation maps A and T_2_ relaxation maps B for the phantom’s inserts from one acquisition in the T_1_ array layer. **c**, **d** Scatter plots of MRF-based relaxation times (*y*-axis) versus nominal relaxation times (*x*-axis) for T_1_ C and in T_2_ up to 1600 ms D and 200 ms E show linear strong correlation for MRF-based relaxation times compared to nominal relaxation times for both T1 and T2, with underestimation for both properties at higher ranges of T_1_ and T_2_. The error bars represent the mean standard deviation within the regions of interest within each phantom’s insert

MRF-based T_1_ and T_2_ relaxation times of the NIST phantom in the T_1_ array layer are shown in Fig. 3. Figure 3a, b depict T_1_ and T_2_ relaxation maps through the center of the phantom identifying the 14 inserts used to estimate the respective relaxometry values. The standard deviation along the mean for seven MRF measurements as a function of T_1_ and T_2_ relaxation times was calculated for each vial and ranged from 0.4 to 6.8 ms and 0.4 to 12.1 ms for T_1_ and T_2_, respectively. Figures 3(C) show the mean measured versus reference relaxometry values for T_1_ and T_2_ up to 1600 ms (D) and 200 ms (E). While both T_1_ and T_2_ MRF-based relaxometry were highly linear correlated (T_1_: *r*^2^ = 0.99; T_2_: *r*^2^ = 0.99 15–200 ms; T_2_: *r*^2^ = 0.97 15 ms–1600 ms), there was a higher agreement for T_1_ compared to T_2_ values and is reflected in the mean absolute percentage error for MRF-based relaxometry which was 11% for T_1_ and 27% for T_2_. The mean absolute percentage error was lower (3% for T_1_ and 14% for T_2_) for the clinically relevant relaxation times from all the regions analyzed in this study (T_1_ range 500–2000 ms and T_2_ range 15–200 ms).

#### In vivo

**Fig. 4:**
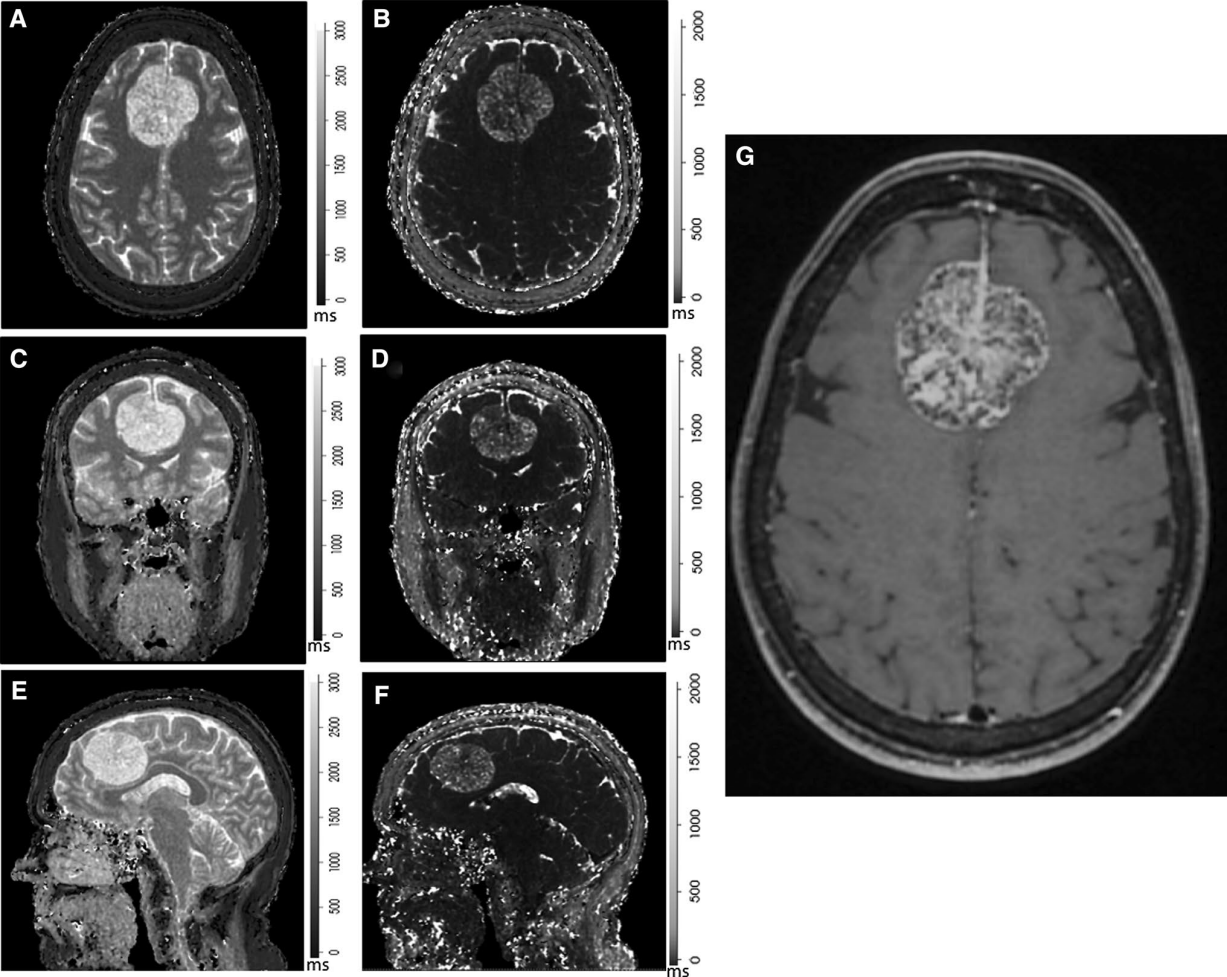
MRF relaxometry maps on axial **a**, **b**, coronal **c**, **d**, sagittal **e**, **f** and T_1_WI post gadolinium **g** in a pathology proven atypical meningioma (Grade II). MRF-derived T_1_-relaxometry (**a**, **c**, **e**) and T_2_-relaxometry (**b**, **d**, **f**) maps show large inter-hemispheric meningioma with T_1_ and T_2_ relaxation times of 2020 ms and 158 ms respectively. **c** Axial post-gadolinium T_1_WI shows the non-homogenous enhancement and higher internal cystic component with resultant longer relaxation times both on T_1_ and T_2_

**Fig. 5:**
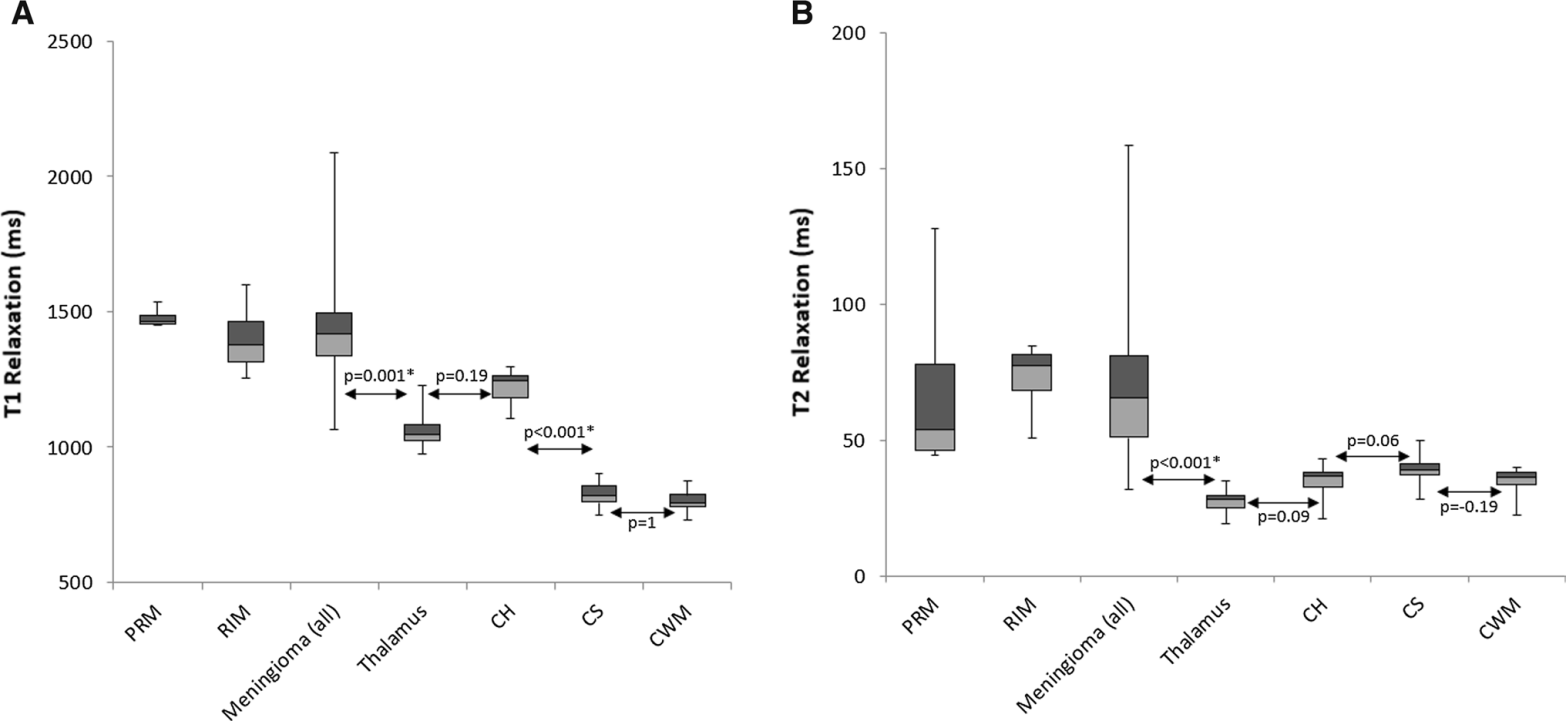
Box and whisker plots for differences in T_1_ (**a**) and T_2_ (**b**) relaxation times between meningiomas and normal brain structures. The longitudinal lines depict the ranges, the light gray box the second quartile, the dark gray the third quartile and the solid horizontal line the median. For the meningiomas, the 25th percentile (T_1_; T_2_: 1335 ms; 51 ms), median (T_1_;T_2_: 1416 ms; 65 ms), 75 percentile (T_1_;T_2_: 1493 ms; 81 ms) were higher compared to all the anatomical structures. The different groups of meningiomas showed similar T_1_ and T_2_ relaxation times. *PRM* partially resected meningioma, *RIM* radiation induced meningioma, *CH* caudate head, *CS* centrum semiovale, *CWM* contralateral white matter. *p < 0.05 connotes statistical significance

Eleven of the 20 patients (mean age of 59 ± 13 years (mean ± SD) were female. The median time between initial diagnosis to MRF imaging was 13 months (0–198 months). For patients with multiple meningiomas (*n* = 4), a single patient had three lesions (2 RIM, 1 PRM) while the remaining three had two lesions each (two patients had two NTM; another patient; two RIM). Grade I meningioma (*n* = 6) had a mean T_1_ ± SD and T_2_ ± SD of 1436 ± 72 ms and 65 ± 34 ms, respectively, while Grade II (*n* = 3) had a mean T_1_ and T_2_ of 1599 ± 388 and 90 ms ± 53. Representative MRF maps obtained from one patient are shown in Fig. 4. Figure 5 shows the box and whisker plots for differences in T_1_ and T_2_ relaxation times between meningioma and normal structures.

Table 1 lists the statistical analysis of MRF-based relaxometry data for all patients. Mean T_1_ relaxation times were significantly longer for meningioma when compared to the thalamus (*p* = 0.001), CS (*p* < 0.001) and CWM (*p* < 0.001). Deep gray matter showed longer T_1_ relaxation compared to white matter, as represented by the CH versus the CS (*p* < 0.001) and CWM (*p* < 0.001) and the thalamus compared to CWM (*p* < 0.001). All other statistical comparisons between anatomical structures were not significant. Mean T_2_ relaxation of meningioma times were longer than the thalamus (*p* < 0.001), CH (*p* < 0.001), CWM (*p* < 0.001). T_2_ relaxation times of the CS was significantly longer than the thalamus (*p* < 0.001). All other pair-wise comparisons for T_2_ were not statistically significant.

**Table 1:** T_1_ and T_2_ MRF-based relaxometry values for all patients

	T_1_ mean (ms)	SD T_1_^1^ (ms)	*p*	T_2_ mean (ms)	SD T_2_^1^ (ms)	*p*
Meningioma (*n* = 25)	1429	202	Reference	69	27	Reference
Thalamus (*n* = 20)	1054	58	0.001*	27	3	< 0.001*
CH (*n* = 20)	1223	52	0.19	35	5	< 0.001*
CS (*n* = 20)	825	42	< 0.001*	29	5	0.15
CWM (*n* = 20)	799	45	< 0.001*	35	4	< 0.001*

*CS* centrum semiovale, *CH* caudate head, *CWM* contralateral white matter

*p < 0.05 connotes statistical significance; values on bold are considered statistically significant

^a^Standard deviation along the mean T_1_ and T_2_ among patients

## Discussion

MRF allows accurate and time-efficient quantitative mapping of MR tissue relaxometry values across various organ systems [11, 14]. In neuroimaging, MRF has been used for evaluation of intracranial tumors [23, 24, 40] and epilepsy [7, 22, 41]. To our knowledge, this is the first study to report on the use of this technique in a whole-brain 3D scheme covering the entire brain in patients with meningioma under routine clinical imaging conditions. Using the interleaved MRF spiral acquisition, isotropic coverage of the whole brain at a resolution of 1mm^3^ per voxel was achieved in less than 5 min of acquisition time (4:38 min: seconds). Validation was obtained using a standardized phantom where three-dimensional MRF-based relaxometry values were linearly correlated with their stated reference values (*r*^2^ = 0.99 for T_1_ and T_2_) over the range of clinically relevant T_1_ and T_2_ relaxometry values.

In the phantom data presented perfect agreement between the calculated and stated relaxometry values was not achieved across the range of T_1_ and T_2_ values tested. This difference was more evident in T_2_ compared to T_1_ estimates and most prominent at the longest T_2_ value (1500 ms) (Fig. 3c–e). Within the context of this study, the impact of these variations is minimal as the in-vivo brain tissue estimates are characterized by T_2_ values of less than 200 ms at 3T [42] and, therefore, expected to be accurately determined using the proposed MRF technique. Furthermore, the relaxometry range at 3T described for peritumoral white matter (including edema), glioblastoma multiforme, low-grade gliomas and metastatic disease is bellow 200 ms [23, 24]. While similar conclusions cannot be made with regards to MRF-based estimates of CSF, which is known to have a T_2_ values of the order of 1000–2000 ms at 3T [43], estimates of CSF were not made and therefore do not impact the results of this study.

There are a few potential reasons for the deviation between the measured and nominal relaxation times for MRF-based T_2_ values. First, the pulse sequence used employs a spoiled gradient echo acquisition scheme resulting in a highly T_1_-weighted MR signal [2, 9]. Second, the sequence does not employ any additional T_2_ preparation pulses to provide greater T_2_ weighting thereby being less sensitive to tissues with long T_2_ relaxation times. Third, the sequence does not account for effects such as magnetization transfer, diffusion and field inhomogeneities which can affect relaxometry estimates [9, 44].

MRF T_1_ and T_2_ relaxation values of the selected normal brain structures in this study are in agreement with previously published results derived from methods using a 2D acquisition [20, 42]. Badve et al. [42] investigated MRF-based relaxation in white matter structures from selected 4–5 brain slices, showing increased WM relaxation with aging. The relaxation range in the WM (700–1000 ms, 50–30 ms; T_1_, T_2_) was similar to our results. Korzdorfer et at [20] studied a 2D-MRF acquisition with seven slices and different field strengths (1.5 T and 3T) in selected brain structures, identifying a longer relaxation for the thalamus and caudate (1200–1400 ms, 40–50 ms; T_1_, T_2_) compared to WM, also consistent with the findings of this investigation. The novelty of our study is the introduction of a whole-brain 3D-MRF acquisition that is able to describe similar results obtained from other studies but with an isotropic 1 mm^3^ resolution allowing the reconstruction of a 512 axial, coronal or sagittal slices. Therefore, the entire brain can be depicted. Importantly, this study was performed on a scanner from a different manufacturer with an acquisition time that is even shorter than those mentioned studies (acquisition length was between 5 [20] and 10 min [45]). The reduced acquisition time allows the proposed sequence to attain robust clinical feasibility and shows that MRF-based relaxation may be manufacturer agnostic.

The use of MRF-based T_1_ and T_2_ relaxometry represents an evolution of the concept of relaxometry as a biomarker of disease in the diagnosis and staging of intracranial masses [24, 42]. As early as 1980s, investigators described using two different spin echo and one inversion recovery pulse sequences to compute relaxation values of meningioma [46, 47]. The study by Komiyama et al. [46] indicated that T_1_ and T_2_ values were greater for meningioma compared to anatomical structures due to higher water content, a finding consistent with the results reported in this work. Similarly, MRF-based relaxometry values for intra-parenchymal brain tumors have been reported to be greater than normal tissue [23]. However, the advantage of MRF-based estimates is reduced acquisition time when compared to conventional T_1_ and T_2_ mapping techniques, simultaneous multi-parameter relaxometry mapping, improved accuracy, repeatability and reproducibility based on phantom experiments [15] and in-vivo studies [20]. It is therefore feasible that MRF could be used as a clinical tool to rapidly and accurately quantify relaxation times as a biomarker of disease for meningiomas as well as other intracranial brain masses [23, 24, 40].

Quantitative MRI acquisitions other than MRF have been proposed to further elucidate the histological features of meningiomas, such as MR elastography and its based-stiffness estimates [34] as a possible presurgical planning tool [48]. In addition, ADC values acquired from DWI have been shown to correlate with aggressiveness of meningioma with lower ADC values indicating more aggressive meningioma [31, 32]. Recently, Zhang et al. correlated longer MRF-based relaxations with softer meningioma consistency after surgical resection, although the stiffness evaluation in this work was subjective [49]. In this case, MRF-based relaxometry depicts quantitative information of meningioma stiffness, but importantly it may be capable of differentiating meningioma based on grade. Peritumoral edema is a feature that may indicate more invasive meningioma [50], which in turn could led to a longer relaxation as analogous to previously published MRF-based correlation of increasing brain glioma tumor grade with prolongation of relaxation times [23, 24]. However, the use of MRF-based relaxation for predicting meningioma grade needs a much larger number of comparisons to design a statistically meaningful analysis than performed in this investigation, and given only three meningiomas were Grade II, we were not able to perform any statistical test for testing differences between Grade I and Grade II tumors. Importantly, surgical and treatment planning of meningioma are influenced by their histological grade [30–32, 48, 51] and if MRF could distinguish this, it could have major clinical applications.

There are several limitations of this study. The sample size was small and was not powered to detect differences between different types of meningioma. While statistical significance was detected between meningioma and brain structures, a larger sample size would likely provide a powered study to further evaluate these differences. Second, the diagnosis of meningioma was based on imaging findings and only nine lesions had histologic confirmation. Ideally, complete classification of meningioma would require histological analysis, radiation status and tumor growth so as-to more accurately correlate with MRF-based relaxometry values.

## Conclusions

Whole-brain 3D-MR fingerprinting relaxometry estimates have strong linear relationship with nominal values under experimental phantom studies for the expected in-vivo brain relaxometry values. Importantly, the short acquisition allows MR fingerprinting to be feasible in a clinical setting where relaxometry properties in meningiomas can be characterized using a high spatial resolution of 1mm^3^ and potentially shortening MRI time when surveillance of those tumors is warranted. Although this analysis was not empowered enough to assess the potential role of MRF-based relaxometry as biomarker of tumor grade, the promising features we described with MR fingerprinting may guide investigations with larger samples.
